# Using nanotechnology to deliver biomolecules from nose to brain — peptides, proteins, monoclonal antibodies and RNA

**DOI:** 10.1007/s13346-021-01086-2

**Published:** 2021-11-03

**Authors:** Mireya L. Borrajo, María José Alonso

**Affiliations:** 1 Center for Research in Molecular Medicine and Chronic Diseases (CiMUS), Universidade de Santiago de Compostela, Av. Barcelona s/n, Campus Vida, 15782 Santiago de Compostela, Spain; 2 Department of Pharmacy and Pharmaceutical Technology, School of Pharmacy, Universidade de Santiago de Compostela, 15782 Santiago de Compostela, Spain

**Keywords:** Nose-to-brain delivery, Intranasal drug administration, Brain delivery, Biomolecules, Nanomedicine, Nanoparticles, Peptides, Proteins, RNA, Cell-penetrating peptides, Targeting ligands

## Abstract

There is a growing number of biomolecules, including peptides, proteins, monoclonal antibodies and RNA, that could be potentially used for the treatment of central nervous system (CNS) diseases. However, the realization of their potential is being hampered by the extraordinary difficulties these complex biomolecules have to reach the brain in therapeutically meaningful amounts. Nose-to-brain (N-to-B) delivery is now being investigated as a potential option for the direct transport of biomolecules from the nasal cavity to different brain areas. Here, we discuss how different technological approaches enhance this N-to-B transport, with emphasis on those that have shown a potential for clinical translation. We also analyse how the physicochemical properties of nanocarriers and their modification with cell-penetrating peptides (CPPs) and targeting ligands affect their efficacy as N-to-B carriers for biomolecules.

**Graphical abstract:**

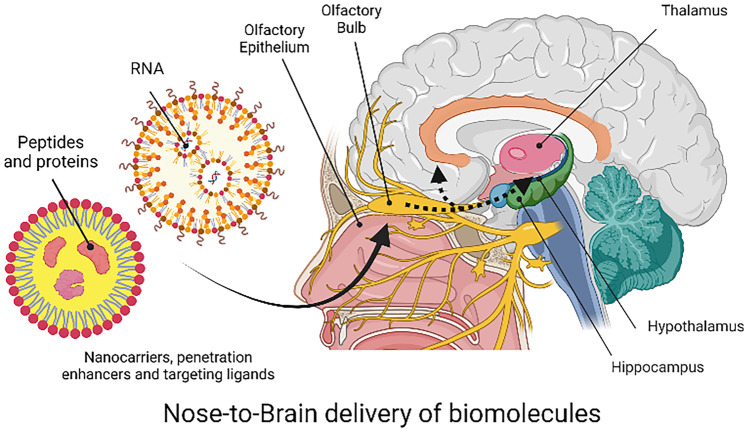

## Introduction

Numerous neurological diseases, including Alzheimer’s disease, Parkinson’s disease, ischemic stroke and multiple sclerosis, among others, have a great prevalence in society. Estimations indicate that the number of people affected by these disorders is growing worldwide, also related to the increased longevity of the population. Apart from the devastating consequences these diseases have in public health, the economic cost associated with their treatment and palliative care is also a matter of great concern [1–3]. The therapeutic effect several biomolecules have shown offers a promising opportunity to treat these diseases. For example, insulin has been investigated at the clinical level (up to Phase 3, after intranasal administration) as a treatment for the cognitive decline in Alzheimer’s disease [4]. A more critical example is the recently approved monoclonal antibody, aducanamab (commercialized as Aduhelm), which was approved for the treatment of Alzheimer’s disease [5–8]. However, despite this recent successful result, their difficult access to the central nervous system (CNS) is an important obstacle for the full exploitation of these biomolecules [9]. The brain is protected by highly restrictive barriers, such as the blood–brain barrier (BBB) and the blood-cerebrospinal fluid barrier (BCSFB) [10–12], which are fundamental for the maintenance of the homeostasis of the CNS, and for the prevention of potential toxic compounds. However, as such, this defence mechanism represents an extraordinary barrier for the transport of drugs to the brain [13, 14]. Moreover, the high metabolic activity of these barriers may contribute to the degradation of the drug molecules transported across them [15, 16]. For decades, researchers have explored a variety of technological approaches to facilitate the transport of biomolecules across these barriers; however, such achievement remains elusive [17, 18]. An alternative approach to reach the brain that is gaining increasing attention makes use of the nose-to-brain (N-to-B) route. Interestingly, the first studies, exploring this modality of administration using a dye in a rabbit model, were carried out in 1937 [19]. From then on, significant knowledge on the mechanisms of transport of molecules across the N-to-B barriers has been generated. As a consequence, it is known that drugs deposited on the olfactory mucosa can be delivered directly into the brain through the olfactory or trigeminal nerves [20, 21]. In line with this knowledge, multiple clinical trials are being conducted, some of them with promising results [22, 23]. Moreover, in the past few decades, nanotechnology has been positioned as a promising strategy for enhancing the N-to-B transport of therapeutic biomolecules [12, 14, 24–29].

Based on this background information, researchers, including members of our group, have published a number of review articles covering different N-to-B delivery strategies [14, 28–31]. Our objective in this article is not to expand the content of previous reviews, but to concentrate on the N-to-B delivery of biologicals, an emerging field that is expected to gain significant relevance in the near future. Hence, we critically analyse the N-to-B drug delivery options for biologicals with emphasis on those based on nanotechnology. Moreover, we disclose our understanding of the critical features for nanosystems to function as carriers to overcome the N-to-B barrier.

## Challenges and barriers for the nose-to-brain delivery of biomolecules

### Pathways for the nose-to-brain delivery of biomolecules

**Fig. 1:**
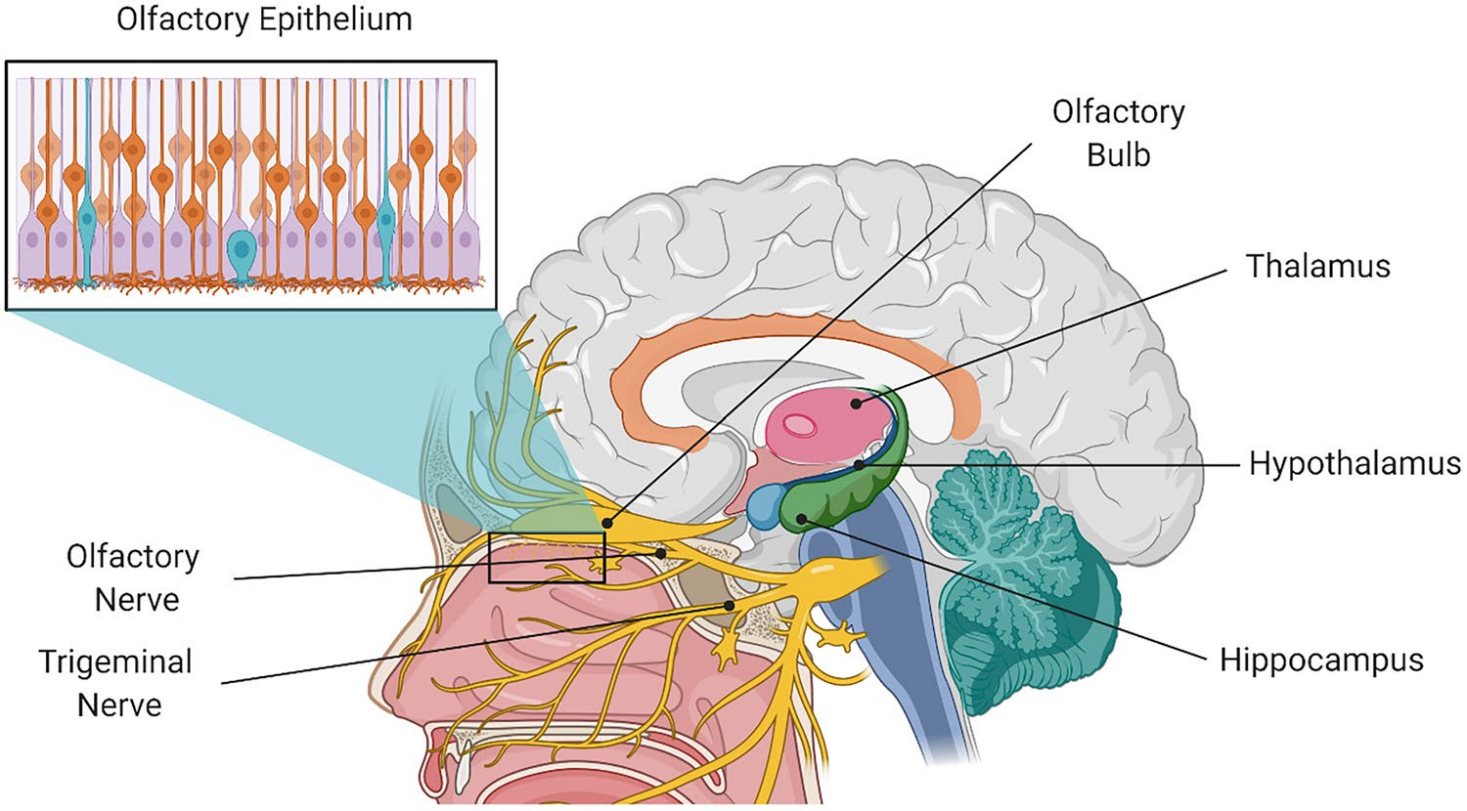
Schematic representation of the olfactory and trigeminal nerve position in the nasal cavity, and pathways to different CNS areas. Created with BioRender.com

The mucosa protecting the nasal cavity can be divided into two main anatomical regions, the respiratory epithelium and the olfactory epithelium (Fig. 1). The respiratory epithelium comprises a major part of the nasal cavity, and it is considered the principal entry from the nose to the blood stream [24, 25]. It presents a high degree of vascularization, receiving its blood supply from the maxillary artery [24]. This mucosa consists of a ciliated epithelium covered by a thick mucus layer [24, 25, 32, 33]. On the other hand, the olfactory epithelium is located on the upper region of the nasal cavity, separated from the CNS by the cribriform plate and the lamina propria and is also protected by a mucus layer [34–36]. This region is highly innervated by the olfactory sensory neurons that connect directly with the olfactory bulb [25, 35]. More detailed information regarding nasal cavity anatomy and physiology can be found in previous reviews [29, 36, 37].

**Fig. 2:**
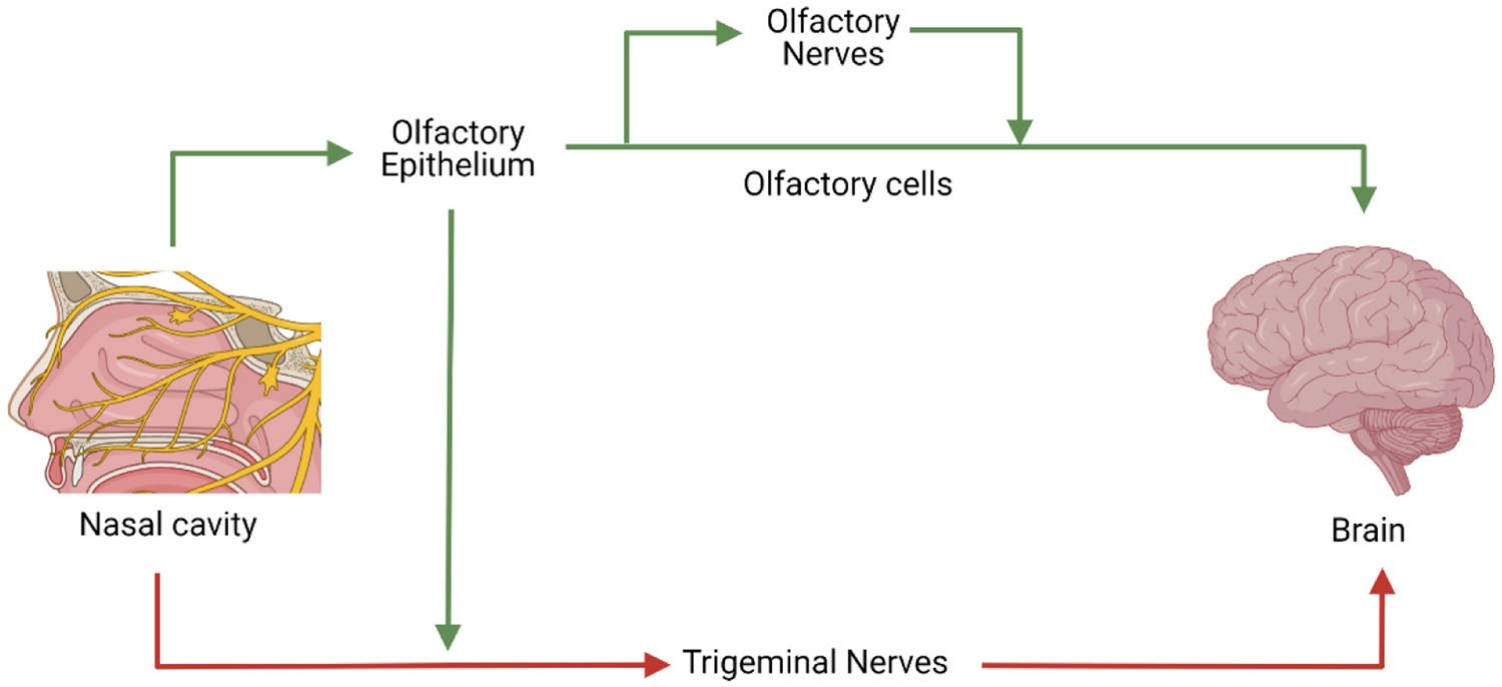
Possible main pathways for N-to-B transport (in green, olfactory pathway; and in red, trigeminal pathway) [29]. Created with BioRender.com

Drugs administered intranasally can reach the brain indirectly, upon systemic absorption through the respiratory epithelium and subsequent transport across the BBB, or directly, across the olfactory epithelium and the olfactory nerves. The indirect transport involving systemic absorption is irrelevant for macromolecules due to the complexity of the multiple barriers associated to the systemic biodistribution and transport across the BBB [27, 38–40]. However, the N-to-B route involving the transport across the olfactory mucosa offers a straight-forward access to the brain, as illustrated in Fig. 2 [25, 27]. Three mechanisms have been described for the N-to-B transport: direct internalization into the olfactory nerve, leading to axonal transport; paracellular transport between epithelial cells and across channels near olfactory nerves; and transcellular transport across cells of the olfactory epithelium [20, 21, 36, 41, 42]. Following these mechanisms, drugs reach the olfactory bulb, from where they can be distributed into the brain [14, 43, 44].

On the other hand, the trigeminal nerve pathway, a less explored route, involves the axonal transport through the trigeminal nerves that innervate both respiratory and olfactory epithelium [45, 46]. The trigeminal nerves synapse at the trigeminal ganglion, entry the brainstem and are directed to the caudal and rostral regions of the brain [45, 47, 48].

### Main challenges in the nose-to-brain transport of biomolecules

Despite the increasing evidence acquired on the potential of the N-to-B drug delivery, the use of this strategy for the delivery of biomolecules must still face significant challenges before it is proved effective. The factors that influence the N-to-B transport of biomolecules include the physicochemical attributes of the biologicals themselves, and the intrinsic anatomical and physiological barriers of the nasal cavity [31, 49].

The main physicochemical properties influencing the passive diffusion of drugs from N-to-B are molecular weight and lipophilicity. Biomolecules, such as peptides, proteins, or nucleic acids, with a size larger than 300 Da and great hydrophilicity, present a lower N-to-B transport when compared to smaller, more lipophilic molecules [46, 50]. Therefore, these molecules are mainly expected to be transported through a receptor-mediated mechanism of transport, such as specific insulin receptors, which are overexpressed in the olfactory bulb and the hypothalamus; or oxytocin receptors, present in high density in the amygdala and hippocampus [51–56].

On the other hand, there are significant anatomical and physiological barriers preventing the access of drugs from nose to brain. First, the access to the olfactory region, located on the posterior and upper region of the nasal cavity, is not easy and requires the use of specialized delivery devices [57–59]. Second, the surface area of the olfactory mucosa is relatively small compared to the whole nasal mucosa [60, 61]. Third, the olfactory epithelium presents long non-mobile cilia that, in combination with the mucus secretion and the presence of metabolic enzymes, hinders the N-to-B transport [12, 61–64]. Fourth, the transport across the epithelium by the different mechanisms highlighted above is highly restricted for water-soluble biomolecules, unless they can use specialized transporters, such as the dopamine active transporter or the glucose transporter, among others [65–68].

Finally, the low dosing volume that can be administered (e.g. a maximal dose of 0.4 mL for humans, a recommended maximum volume of 0.03 mL for mice) is a limitation when using this route and implies the need for highly optimized formulations if a sufficient dose to produce therapeutic effects is to reach the brain [69, 70].

## Clinical scenario of nose-to-brain transport of biomolecules

**Fig. 3:**
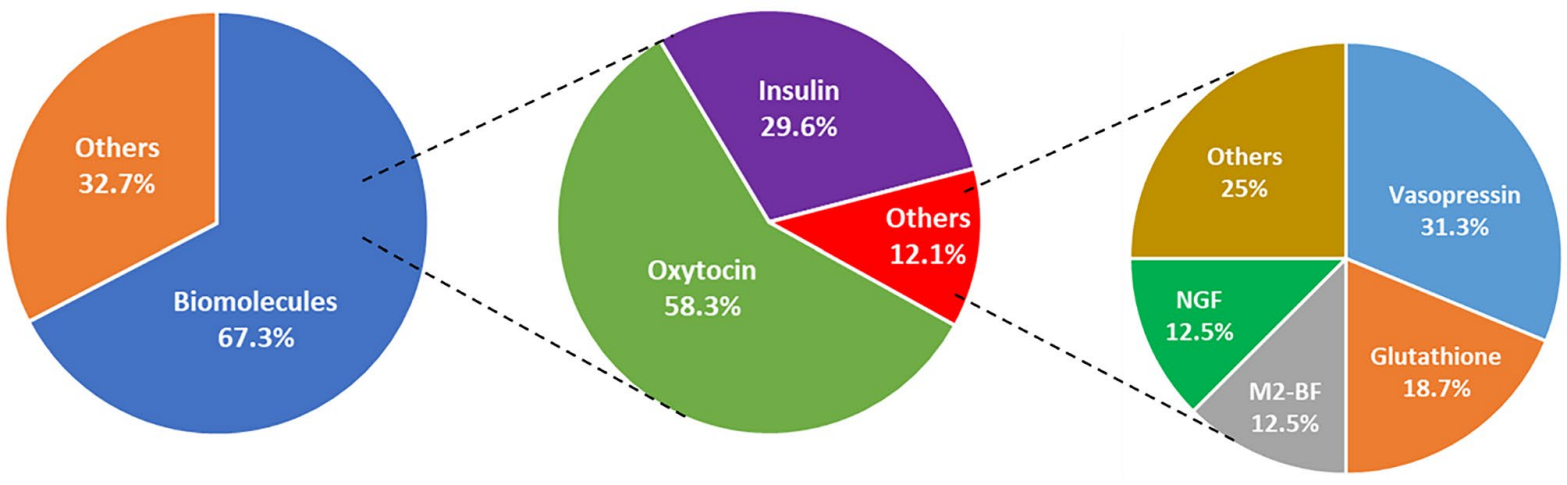
Summary of the clinical trials involving N-to-B drug delivery of biomacromolecules

The clinical trials, specifically aimed at the N-to-B delivery of biomolecules, collected in a ClinicalTrials.gov database, are summarized in Fig. 3 [71]. Among the 196 clinical trials analysed, more than 67% refer to the administration of biomolecules in the form of a simple aqueous solution. The most clinically tested biomolecule is oxytocin, currently in a Phase 4 study for the indication of autism spectrum disorder and schizophrenia, in Phase 3 for the treatment of Prader-Willi syndrome (PWS) and in Phase 2 for the treatment of dementia and obesity.

The intranasal administration of an insulin aqueous solution has also been widely studied in up to Phase 2/3 clinical trials, as a therapy for Alzheimer’s disease, mild cognitive impairment, diabetes, insulin resistance, and Parkinson’s disease, among other conditions. Regarding the indication of Alzheimer’s disease, initial studies reported improvements in memory and changes in Aβ45 levels [72]. However, subsequent clinical trials were not conclusive for this indication [4, 73]. Additional clinical trials are currently being conducted to elucidate the correlation between administering intranasal insulin and both Alzheimer’s disease and mild cognitive impairment. Other highly prevalent diseases extensively studied in clinical trials are diabetes and insulin resistance. Interestingly, clinical studies with participants who presented high hypothalamic insulin sensitivity demonstrated that the insulin performance in the brain enhanced pancreatic insulin secretion [74, 75].

Another relevant biomacromolecule currently in clinical trials is vasopressin. Mainly used for social psychology and communication, and pain perception, it shows promising results in an early phase 1 clinical trial [76–78].

To conclude, more than 130 clinical trials with a diversity of biomolecules have been carried out over the last 20 years. However, so far, only oxytocin has reached the final phase 4 clinical trial. In this scenario, it is becoming apparent that an improvement on the access of these biomolecules to the brain is necessary to achieve conclusive results about their potency in the treatment of different brain conditions.

## Technological approaches for nose-to-brain delivery of biomolecules

**Fig. 4:**
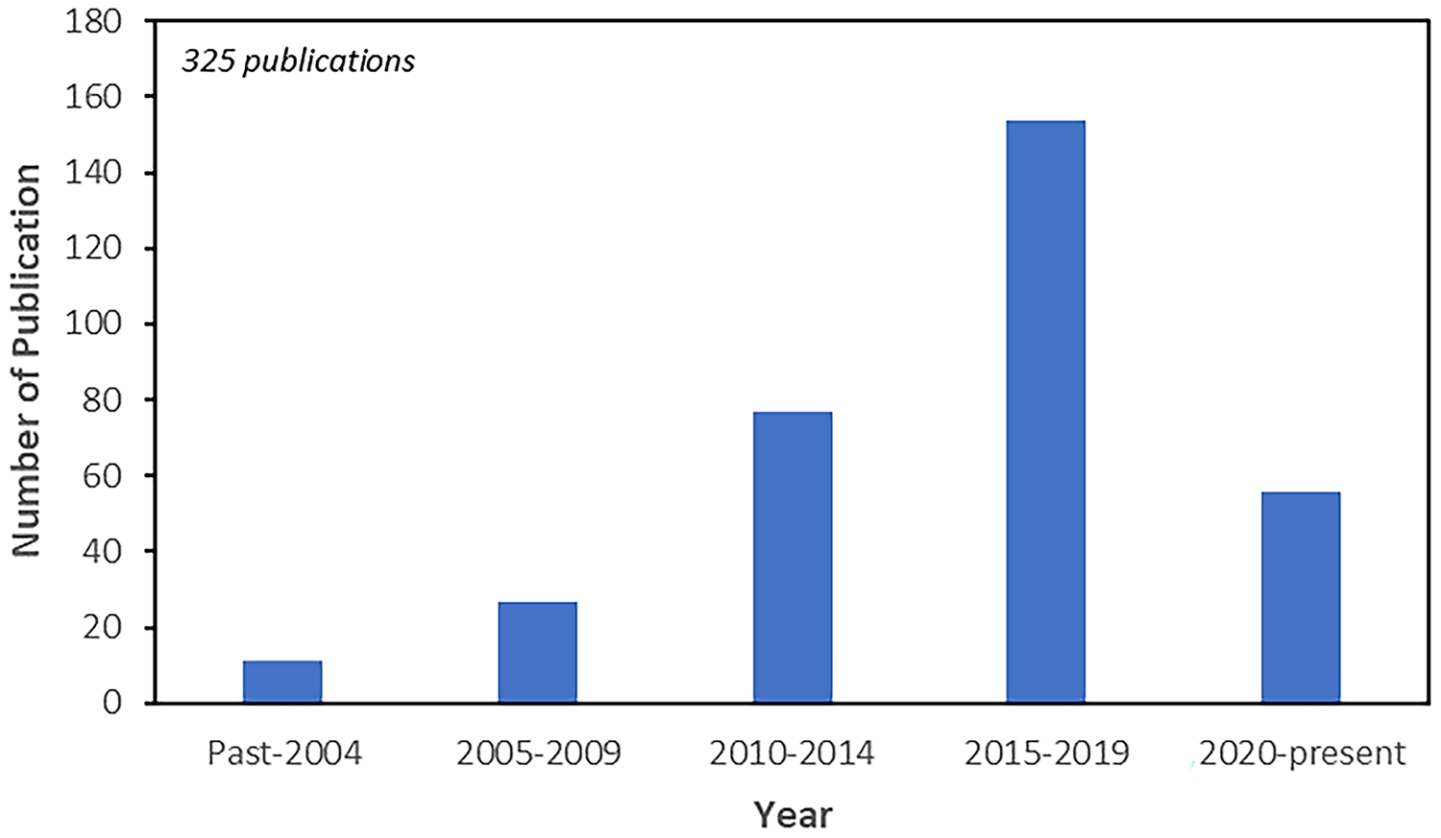
Number of publications of nose-to-brain administration of biomacromolecules (Scopus and PubMed database. Keywords: ‘nose-to-brain + peptide’, ‘nose-to-brain + protein’, ‘nose-to-brain + RNA’)

The increasing interest on the N-to-B delivery of biomolecules is exemplified not only by the significant number of clinical trials being performed, but also by the numerous preclinical studies published presenting evidence of the N-to-B transport (Fig. 4) [79, 80]. A significant increase in the number of publications has been observed from 2010 henceforth. Although the majority of the studies reported refer to simple aqueous solutions of biomolecules, there is an increasing number of publications on delivery strategies for enhancing the N-to-B transport, including penetration enhancers, delivery carriers or a combination of both [29, 31, 44, 81–84].

In the following section, we critically analyse the formulation and delivery strategies explored for the N-to-B delivery of biomolecules.

### Mucoadhesion and penetration enhancement

The use of mucoadhesive polymers has been explored as a way to increase the contact of drugs with the olfactory mucosa and their subsequent diffusion across the olfactory epithelium [12, 25, 32]. However, the enhancement of N-to-B transport of large molecules by use of bioadhesive materials has not been fully validated and only a few examples illustrate this potential mechanism. For example, some authors have reported that the chemical conjugation of hyaluronate, a derivative of hyaluronic acid, with FG loop peptide, a neural cell adhesion molecule-mimetic peptide that is able to decrease the oxidative stress in ischemic episodes, protected the peptide against enzymatic degradation and enhanced its N-to-B delivery [85]. This conclusion was based on the fact that, when using a dose 20 times lower, the hyaluronate conjugate treatment resulted in an infarcted brain area similar to the one shown after treatment with the free FG loop peptide. The authors attributed this positive result to the mucoadhesive properties of hyaluronate; however, the mechanism underlying the transport of the peptide linked to HA remains unclear.

Another mucoadhesive polymer that has been investigated for its potential to increase the N-to-brain access to biomolecules is chitosan, a polysaccharide extensively studied as mucoadhesive and cell penetration agent for N-to-B delivery of small molecules [86, 87]. For example, solutions of chitosan and brain-derived neurotrophic factor (BDNF) reported a 13-fold increase in the accumulation of the protein in the hippocampus [88]. This increased accumulation has been ascribed to the mucoadhesive and penetration enhancement capacity of chitosan.

Overall, while the use of mucoadhesive polymers has been largely reported as a way to enhance the retention of small molecules in the nasal cavity, the translation of the enhanced retention into enhanced adsorption has not been sufficiently validated for biomolecules. This is understandable as the retention of the molecules at the mucosal barrier does not guarantee their subsequent transport to the brain.

The use of penetration enhancers, specially cell penetration peptides (CPPs), is receiving an increasing attention in N-to-B delivery [26, 89]. CPPs are short, positive charge peptides, able to penetrate cellular barriers and facilitate the internalization of co-administered drugs, without the need of interactions with specific receptors [90–92]. Among them, penetratin or pAnt(43–58), a 16-amino-acid peptide that corresponds with the third helix of the Antennapedia homeodomain, a homeoprotein of *Drosophila melanogaster*, has been the most frequently used [93–95]. Penetratin has two isomers with different cell-penetrating properties: L-penetratin and D-penetratin. In a study where both isomers were nasally administered together with radio-labelled insulin to rats, the levels of insulin detected in the anterior portion of the CSF were higher for L-penetratin [96]. This result was further confirmed in Alzheimer’s disease model mice, where co-administration of insulin with L-penetratin reported slower memory loss progression in comparison with the co-administration of insulin with D-penetratin or with the single administration of insulin [97]. Furthermore, differences between both isomers were determined after being co-administered with exedin-4 in Alzheimer’s disease model mice: only L-penetratin was able to enhance the N-to-B delivery of exedin-4 [98].

In other studies, CPPs were covalently linked to the biomolecule of interest. For example, low molecular weight protamine (LMWP) was linked to various proteins, i.e. bovine serum albumin (BSA), horseradish peroxidase (HRP) and β-galactosidase. The resulting protein conjugates were found to have a greater access to the brain, as compared to the non-conjugated biomolecules [99]. Similarly, the conjugation of a nine-arginine peptide to Green Fluorescent Protein (GFP) was reported to facilitate the delivery of GFP to the brain [100].

Finally, surfactants have also been proposed as excipients to increase the permeation of molecules across the olfactory mucosa. For example, Pluronic® P85 fused to the protein leptin was reported to enhance its transmembrane penetration and resulted in a higher activation of leptin receptors in the brain than when free leptin was used [101]. Similarly, the surfactant *n*-tridecyl-β-D-maltoside was also found to increase the permeation of the neuropeptide hexarelin to the brain following nasal administration [102].

Overall, these studies are an indication of the potential interest of penetration and permeation enhancers in combination with biomolecules for improving the N-to-B transport of these biological drugs.

### Nanotechnological approaches

**Fig. 5:**
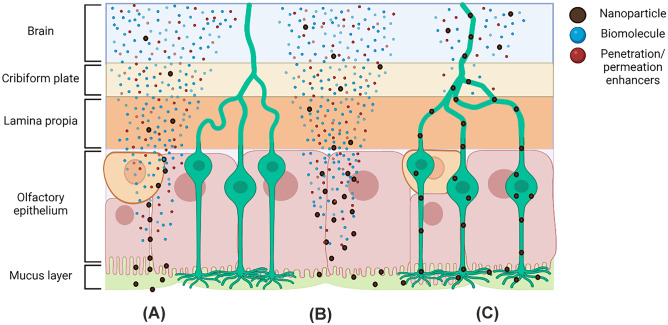
Schematic representation of the possible mechanisms of transport of nanoparticles (round black), drug molecules (blue spots) across the different barriers. Nanoparticles containing drugs and penetration enhancers may cross the olfactory epithelium by a paracellular (**A**) or transcellular pathway (**B**) and may, or may not, release drugs and penetration enhancers (red spots) in their way to the brain. Nanoparticles may be taken up by axons and undergo intra-axonal transport into the olfactory nerve (**C**). Nanoparticles crossing the multiple barriers without releasing their cargo are adequate for RNA delivery, whereas nanoparticles releasing the drug molecules and penetration enhancers at the different levels may be adequate for the delivery of proteins and peptides. The transneuronal transport (**C**) is expected to play a significant role in the delivery of RNA-loaded nanocarriers, whereas the transepithelial transport maybe also be adequate for peptides and proteins. Created with BioRender.com

Recently a number of nanotechnologies have been investigated for their capacity to enhance the transport of biomolecules across the olfactory mucosa. Different transport mechanisms can be followed by nanoparticles and their associated molecules when travelling from nose to brain (Fig. 5). These hypothetical mechanisms are as follows: (A) nanoparticles containing drugs and penetration enhancers may cross the olfactory epithelium by a paracellular pathway and may, or may not, release drugs and penetration enhancers in their way to the brain; (B) nanoparticles containing drugs and penetration enhancers may cross the olfactory epithelium by a transcellular way and may, or may not, release drugs and penetration enhancers in their way to the brain; and (C) nanoparticles may be taken up by axons and undergo intra-axonal transport into the olfactory nerve. Nanoparticles crossing the multiple barriers are adequate for RNA delivery, whereas nanoparticles releasing the drug molecules and penetration enhancers at the different levels may be adequate for the delivery of proteins and peptides. This is because the site of action of the peptides and proteins drugs may be located at the extracellular level. However, in the particular case of nucleic acids, they need to be protected while being transported until their internalization inside the brain cells.

The most common transcellular (B) and intra-neuronal (C) approach that nanoparticles undergo to access the different protective layers of the brain is transcytosis. This phenomenon may involve adsorptive or charge-dependent mechanisms; or receptor-mediated internalization, being this last method more specific and efficient to target the CNS. This mechanism of transport would allow the nanoparticles to undergo through endocytosis, intracellular vesicular trafficking and exocytosis inside the different cellular barriers, aiming to reach their side of action [103–105].

**Fig. 6:**
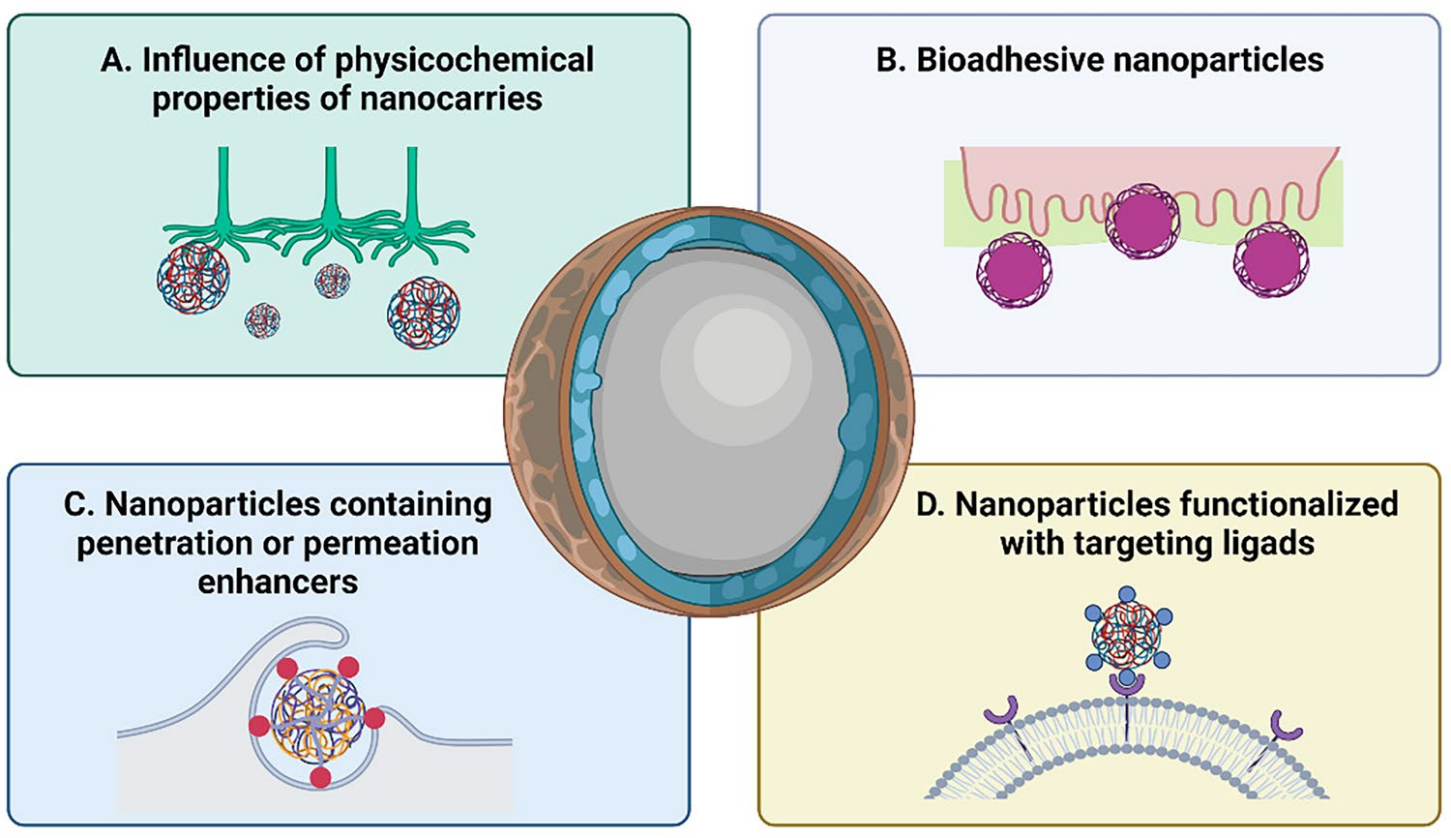
Nanotechnological approaches for the N-to-B delivery of biomolecules. Incorporation of biomolecules (e.g. peptides, proteins and nucleic acids) into different nanosystems can enhance their effective N-to-B transport, which is driven by (**A**) the physicochemical properties of the nanocarriers; (**B**) their bioadhesive nature; (**C**) their surface modification with different permeation or penetration enhancers; or (**D**) their surface functionalization with targeting ligands. Created with BioRender.com

The capacity of nanoparticles to work as carriers for the transport across the different barriers is obviously dependent not only on their physicochemical properties but also on their surface composition, which may influence their bioadhesive behaviour, penetration properties or endocytic uptake (Fig. 6). Below we describe critical information that may guide subsequent advances in the development of N-to-B nanomedicines.

#### Influence of physicochemical properties of nanocarriers

Even though the literature does not give a clear guidance in terms of which physicochemical properties are the most suitable for enhancing the N-to-B transport, it is reasonable to assume that particle size and surface charge may have a fundamental role in the ability of nanocarriers to reach the CNS. Regarding particle size, it is generally accepted that the physiological characteristics of the N-to-B pathways determine the critical size for the nanoparticles (NPs) to be transported through the N-to-B pathway. For example, with regard to the trans-neuron pathway, given that the axons diameter in humans is 100–700 nm, it has been hypothesized that carriers larger than this would not be able to use this route of transport. Based on the fact that the dimension of axons varies between 100 and 200 nm in different animal species [29, 36, 43, 106], the majority of the nanocarriers disclosed for N-to-B delivery have been designed to have a size between 50 and 150 nm (Tables 3–5). However, as the trans-neuron transport is not the only N-to-B pathway, other nanocarriers with a mean size of 270 nm, or even 440 nm, have also shown some positive results in in vivo studies [107–109]. For example, using polysorbate-80 coated polystyrene NPs, it was observed that particle diameters of 100 nm showed more than a fourfold greater brain uptake than the same system with a 180 nm mean size. In a separate study, different sizes of polyethylene glycol-polylactic acid (PEG-PLA) NPs were developed; according to their results, mean diameters of 100 nm showed greater brain accumulation and, in consequence, enhanced therapeutic effect, than nanosystems of 500 nm, in an epilepsy rat model after intranasal administration [110]. In a more recent study on the biodistribution of nanoemulsions with different diameter, it was concluded, using fluorescence imaging, that nanosystems with a diameter of 100 nm were capable to follow both olfactory and trigeminal pathways, while nanocarriers with a larger particle size were not detected on the olfactory bulb [111]. Finally, a report aimed at exploring the influence of the particles chitosan-coated polystyrene NPs (160 and 280 nm) showed that, irrespective of their size, their transport to the brain was negligible [112].

A literature analysis of the influence of the surface charge on the capacity of nanocarriers to overcome the N-to-B barrier leads to the conclusion that it is the chemical composition of the surface, rather than its charge, the main factor influencing the interaction and transport of nanoparticles across the olfactory mucosa. In fact, different nanocarriers with surface charges from + 57 to − 30 mV have shown significant efficacy in enhancing N-to-B delivery of different biomolecules [108, 113–115]. For example, chitosan (positive charge)- and polysorbate-80 (negative charge)-coated polystyrene NPs were investigated for their transport across the murine olfactory epithelium. The fluorescence imaging results showed that negatively charged nanocarriers were internalized in the olfactory epithelium in a greater extent than the positively charged ones. This might be attributed to the retention of chitosan nanocarriers in the mucus layer of the nasal cavity [43, 112]. In a more recent study, the brain distribution of fluorescent positive (chitosan-coated poly(lactic-glycolic acid) (PLGA) NPs) and negative (PLGA NPs) nanocarriers was followed after intranasal administration. Even though both NPs were found in different areas of the brain, the trigeminal pathway seems to be the area mainly responsible for the transport of positively charged nanosystems while the negative NPs seem to be transported primarily by the olfactory pathway. Importantly, these differences on the main N-to-B pathway followed could also be attributed to the difference in particle size observed in positive (213 nm) and negative (118 nm) NPs [116]. Further studies comparing nanocarriers with different surface charges but maintaining similar particle diameters should be performed to confirm this hypothesis.

Overall, it is clear that the physicochemical properties of nanosystems influence the success of N-to-B delivery of biomolecules, but no clear conclusions can be reached regarding the limits in particle size or surface charge that are more favourable to enhance the N-to-B transport.

#### Bioadhesive nanoparticles

Although the interest on increasing the bioadhesion or mucoadhesion of nanocarriers to enhance the N-to-B delivery of biomolecules is still being elucidated, examples of the incorporation of adhesive regents into nanosystems to facilitate N-to-B transport of biomolecules are highlighted in the literature.

Only a few manuscripts have reported the potential positive role of mucoadhesion in the N-to-B transport of nanostructures. For example, some studies reported the incorporation within in situ-forming gels of radioactively labelled siRNA complexed into dendriplexes. After intranasal administration of these Carbopol-containing dendriplexes, the radio-labelled accumulation in the brain detected was greater than the one obtained when using dendriplex or free siRNA [117]. Other materials have been used for the development of nanogels, such as poly(*N*-vinyl pyrrolidone) (PVP), that seem to depict certain mucoadhesive properties [118]. PVP-based nanogels have been studied for the delivery of insulin to the brain. After intranasal administration in mice, twofold greater fluorescent levels were detected in the brain, compared with the N-to-B administration of free insulin [119].

Despite these results, it is still unclear what the role of bioadhesion or mucoadhesion on enhancing the delivery of biomolecules to the brain may be. As mentioned in this review article, mucoadhesion properties increase the residence time in the nasal cavity and the further delivery of small molecules to the brain, but the same principle does not apply to biomolecules. In fact, as indicated for chitosan, the mucoadhesive properties of nanoparticles and gels may promote their retention at the mucus level. Further studies need to be performed in order to conclusively elucidate the role of bioadhesion and mucoadhesion on the nanocarrier-mediated N-to-B transport of biomolecules.

#### Nanoparticles containing penetration or permeation enhancers

Based on the observed fact that the co-administration of permeation enhancers or CPPs with biomolecules can enhance their N-to-B transport, some authors have proposed the idea of incorporating CPP as a constituent of nanoparticles. For example, as shown in Table 1, a few studies have used a covalent linkage of CPPs to the nanoparticles. In some cases, a 13 amino acid truncate version of the trans-activator of transcription (Tat) protein of human immunodeficiency virus (HIV-1) was used [120–122]. The benefit of Tat in terms of enhancing the N-to-B transport was assessed upon covalent linking to insulin-loaded PLGA NPs. The brain accumulation of fluorescently labelled Tat-modified NPs, measured by fluorescence microscopy, was 6 times greater than the one obtained using the non-modified NPs [123]. The same approach was applied to PEG-poly (ɛ-caprolactone) (PEG-PCL) nanomicelles, loaded with different types of siRNA. In all cases, both the therapeutic effect and the brain biodistribution were greater when nanomicelles were modified with Tat than with plain nanomicelles [124–126]. The influence of the Tat density on the surface of PEG-PLA nanomicelles in their transport across the olfactory mucosa was also explored in a different study. The authors determined that micelles with a Tat surface density of 10% were able to get through trigeminal nerves at a higher extent than those with a lower Tat density. Nanosystems were also observed in the olfactory nerves, but their presence was mainly attributed to their small size, because regardless of the Tat surface density, the mean diameter size was 20–35 nm [127].

**Table 1:** Examples of selected nanosystems modified with CPPs for N-to-B delivery

Nanosystem	Cell-penetrating peptide	Animal model	Ref
PLGA NPs	Tat	Mice	[123]
PEG-PCL nanomicelles	Tat	Rat	[124–126]
PEG-PLA nanomicelles	Tat	Rat	[127]
PGA-PLA NPs	LMWP	Rat	[128]
Nanomicelles	CH2R4H2C	Rat	[129]
Nanocomplexes	C12-r8	Mice	[130]

*LMWP* low molecular weight protamine, *PEG-PCL* PEG-poly(ɛ-caprolactone), *PEG-PLA* PEG-polylactic acid, *PLGA* poly(lactic-glycolic acid), *PEG-PLA* PEG-polylactic acid

A different CPPs mentioned above, LMWP, was also conjugated to fluorescently labelled PEG-PLA NPs. The modified NPs showed a greater brain distribution into cerebrum, cerebellum, olfactory tract and olfactory bulb, also including olfactory and trigeminal nerves, in comparison with non-modified NPs [128]. These results correlate with previously discussed studies where LMWP were directly fused with different proteins, which enhanced their N-to-B transport [99].

Another CPP used to study the brain biodistribution of fluorescent-dextran-loaded nanomicelles upon nasal administration was a basic peptide composed of arginine, histidine and cysteine (CH2R4H2C). The micelles were based on a hydrophobic derivative (stearyl modification) or on a hydrophilic derivative (PEG-PCL modification) of the CPP. Micelles made with C16-CPP showed accumulation in the olfactory bulb, with no relevant distribution to other brain areas; whereas micelles made of CPP-PEG-PLC showed a broad distribution in the brain. Even though authors concluded that the brain distribution of the hydrophilic derivative suggested that their uptake is mediated by both the olfactory and the trigeminal pathways, this could be also be explained by the positive effect of the PEGylation of the polymer leading to an enhanced diffusion across the brain stroma [129].

Our group has also used a hydrophobic derivative of octarginine (C12-r8) to form nanocomplexes with miRNA. The resulting nanocomplexes were subsequently enveloped with an amphiphilic polymer, poly(glutamic acid)-PEG (PGA-PEG). These enveloped nanocomplexes (ENCPs) were found to transport miRNA to the brain and to modulate its mRNA targets [130].

Importantly, despite the positive results in terms of enhanced transport achieved with the incorporation of CPPs to the nanocarriers, it should be kept in mind that the amount of these compounds may need to be limited in order to avoid potential immunogenicity problems [131, 132].

In addition to the so-called CPPs, cationic polymers, notably chitosan, have also been explored for their penetration-enhancing properties in the form of nanoparticles. Chitosan nanoparticles have been used for the N-to-B delivery of different kinds of siRNA as commented in the following section [113, 133]. Hybrid nanocarriers, combining chitosan with manganese or with gold NPs (AuNPs), have also been proven to enhance the delivery of multiple types of RNA to different brain areas [134, 135]. Moreover, chitosan has been used as a coating material for pre-formed nanocarriers. For example, different studies using nanostructured lipid carriers coated with chitosan and containing human insulin-like neurotrophic growth factor-I (hIFG-1) or glial cell-derived neurotrophic factor (GDNF) reported an important accumulation of these factors in the brain, as well as a significant therapeutic effect. Unfortunately, these studies failed to use the adequate comparators (nanocarriers without chitosan) and, hence, the specific role of chitosan in these formulations could not be assessed [136, 137]. Trimethyl-chitosan (TMC), a chitosan derivative with enhanced adhesion properties, was used in the form of a nanocomplex with the neuropeptide, leucine-enkephalin (Leu-Enk). After intranasal administration, the increased delivery to the brain was explained by the electrostatic interaction of the cationic nanoparticles with the brain capillaries, although this qualitative information is very speculative and obtained from a microscopic observation of sections of the mouse brain. The mechanism of action of chitosan for the enhancement of permeation of nanocarrier is still to be elucidated [107].

In addition to these biomaterials, either peptides or polymers, recognized as penetration or permeation-enhancing agents, we must note that the use of surfactants is expected to play a role in the penetration of the nanoparticles into the olfactory mucosa. Overall, these reports are an indication of the potential interest of combining CPPs and other penetration enhancers with NPs.

#### Nanoparticles functionalized with targeting ligands

Covalent modification of the nanocarriers’ surface with targeting ligands offers an interesting possibility to optimize the N-to-B delivery, as summarized in Table 2. According to our records, the first targeting ligand to be incorporated onto nanocarriers was wheat germ agglutinin (WGA), a lectin with specific binding to *N-*acetyl-D-glucosamine and sialic acid, present on the surface of the epithelial cells of the olfactory mucosa [138–140]. Initial studies on the functionality of PEG-PLA NPs containing vasoactive intestinal peptide reported a significantly higher brain accumulation, in comparison with the one found using unmodified NPs [141]. Further studies intended to elucidate the brain biodistribution of these nanocarriers, indicated that the accumulation was particularly noticeable in the olfactory bulb, which suggested their transport through the olfactory pathway [142]. WGA-functionalized PEG-PLA nanoparticles were also applied to the N-to-B transport of miRNA-132. The results obtained using fluorescence imaging showed an enhanced accumulation of the functionalized DiR-labelled nanocarriers, both in Alzheimer’s disease mice and ischemic rat models [143]. WGA-functionalized PEG-PLA NPs were also applied to the N-to-B delivery of the NR2B9c peptide, in an ischemic stroke rat model [144]. This study found a greater reduction on the infarcted area when compared to the one obtained for the unmodified nanocarrier.

**Table 2:** Examples of selected nanosystems modified with targeting ligands for N-to-B delivery

Nanosystem	Targeting ligand	Animal model	Ref
PEG-PLA NPs	WGA	Mice/Rats	[141–143]
PEG-PLGA NPs	WGA	Rats	[144]
PEG-PLGA NPs	STL	Rats	[146, 147]
PEG-PLGA NPs	OL	Mice/Rats	[149, 150]
Cubosomes	OL	Rats	[151]
PEG-PCL NPs	Lf	Mice	[155]
PEG-PLGA NPs	RVG29	Rat	[157]
Au-Fe_2_O_3_ NPs	T7	Mice	[134]
AuNPs	D1	Rat	[158]

*Au-Fe*_*2*_*O*_*3*_ gold-iron oxide, *AuNPs* gold nanoparticles, *Lf* lactoferrin, *NPs* nanoparticles, *OL* odorralectin, *PEG-PCL* PEG-poly (ɛ-caprolactone), *PEG-PLA* PEG-polylactic acid *PEG-PLGA* PEG-poly(lactic-glycolic acid), *PLGA* poly(lactic-glycolic acid), *RVG29* rabies virus glycoprotein, *STL* *Solanum tuberosum* lectin, *WGA* wheat germ agglutinin

Besides WGA, other lectins have been proven to facilitate the N-to-B delivery of biomolecules by modifying the surface of the nanocarriers. For example, *Solanum tubersum* lectin (STL), a glycoprotein that binds to *N-*acetyl-D-glucosamine of the nasal cavity epithelium [140, 145], has been reported to enhance the transport of PEG-PLGA NPs loaded with a fluorescent probe to the brain. Indeed, the brain uptake measured by fluorescence imaging was 2.5-fold greater compared to the unmodified NPs [146]. Additionally, the same group loaded basic fibroblast growth factor (bFGF) into PEG-PLGA NPs functionalized with STL. This study showed a threefold increase of the AUC of bFGF, when compared to unmodified nanocarriers [147].

Despite these positive results, lectins have been reported to exhibit a certain immunotoxicity and this fact has encouraged researchers to explore small lectin-like peptides. For example, Odorranalectin (OL), a 1.7 kDa small peptide originally obtained from frog skin, which was found to specifically bind to L-fucose overexpressed in the olfactory epithelium [145, 148], was used to functionalize PEG-PLA nanoparticles. Using fluorescence imaging, it was observed that the transport of fluorescently labelled functionalized NPs was superior than the one obtained with the unmodified nanocarrier [149]. Moreover, the same authors analysed the potential of this system for the N-to-B delivery of the peptide urocortin, which is indicated in the treatment of neurological disorders. The in vivo results obtained in a Parkinson’s disease mice model showed a neurological recovery, which was associated to the significant transport of urocortin [150]. Similar brain biodistribution results were obtained with PEGylated cubosomes functionalized with OL and loaded with Gly14-Humanin (S14G-HN). An increase in the drug concentration in the brain as well as a significant enhancement of the therapeutic effect after the surface modification was observed [151].

One of the most frequently used targeting moieties that has been incorporated on the surface of nanoparticles is the glycoprotein lactoferrin (Lf), a ligand of the lactoferrin receptor (LfR) highly expressed in brain endothelial cells and neurons [152–154]. The results of an in vivo fluorescence imaging study using fluorescently labelled PEG-PCL NPs indicated that Lf enhanced the accumulation of the NPs in the brain. Moreover, when these Lf-functionalized PEG-PCL NPs were applied to the N-to-B delivery of NAP peptide, a greater neuroprotective effect after ligand modification was reported [155].

Other peptidic moieties have been used for enhancing N-to-B delivery. A small portion of the rabies virus glycopeptide (RVG29), responsible for the cellular entry and virus fusion, efficiently binds to the nicotinic acetylcholine receptor (NAchR), that is present in CNS cells [156]. It has been proven that RVG29 in combination with PEG-PLGA NPs significantly enhances brain delivery of miRNA-124. Curiously, both RVG29 and PEG are needed to obtain the highest miRNA accumulation and therapeutic effect [157].

Inorganic NPs have also been modified with different targeting peptides. T7 peptide (Ac-Cys-His-Ala-Ile-Tyr-Pro-Arg-CONH_2_) in combination with gold-iron oxide NPs (Au-Fe_2_O_3_ NPs) showed greater accumulation in the brain than unmodified nanosystem. The same result was obtained when using a modification of D1 peptide (Gln-Ser-His-Tyr-Arg-His-Ile-Ser-Pro-Ala-Gln-Val) of gold NPs (AuNPs), even though comparison with the unmodified carrier was not reported [158].

More relevant studies have been developed to check the efficacy of targeting ligands. These studies were mainly performed using fluorescent or radio-labelled probes, or small molecules [159–163]. They concluded that specific ligands, i.e. lectins, glycoproteins, and glycopeptides, with affinity for epithelial cells or neurone receptors enhance the N-to-B transport of nanoparticles loaded with biomolecules.

## Illustrative nanotechnologies for nose-to-brain delivery of biomolecules

As reported in the previous section, different types of nanocarriers have been designed to improve the protection of biomolecules from degradation and their N-to-B transport [164–166]. Here, we describe a number of selected nanotechnology-based formulations containing specific biomolecules (peptides, proteins and nucleic acids) that were found to be effective in the treatment of neurological diseases.

### Peptides

The delivery of peptides to the brain is a topic that has attracted increasing attention over the past decades, due to their pharmacological value for the treatment of CNS diseases, including neurodegenerative diseases, cancer or ischemic strokes [167–169]. Nanotechnology offers the possibility to overcome the challenges that peptides face to achieve N-to-B transport (Table 3) [28, 170].

**Table 3:** Overview of selected nanocarrier systems for peptide N-to-B delivery

Nanosystem	Peptide cargo	Disease	Size (nm)	Z-Pot (mV)	Targeting molecule	Animal model	Ref
PEG-PLA NPs	VIP	AD	~ 120	-	WGA	Mice	[141]
PEGylted cubosomes	S14G-HN (humanin derivative)	AD	93	− 14	OL	Rats	[151]
PEG-PCL	NAP	AD	88	− 24	Lf	Mice	[155]
PVP nanogels	Insulin	AD	90	− 25	-	Mice	[119]
PEG-PLGA	Urocortin	PD	115	− 20	OL	Rats	[150]
Gelatin NLC	SP	PD	172	− 30	-	Rats	[114]
D,L-PLA NPs	TRH	Epilepsy	108	-	-	Rats	[110]
PEG-PLGA	NR2B9c	Ischemia	139	− 23	WGA	Rats	[144]
*N*-trimethyl chitosan NPs	Leucine-enkephalin	Pain	443	+ 15	-	Mice	[107]
Oil-in-water nanoemulsion	CsA	-	272	+ 57	-	Rats	[108]

*AD* Alzheimer’s disease, *CsA* cyclosporine-A, *Lf* lactoferrin, *NCL* nanostructured lipid carriers, *NPs*, nanoparticles, *OL* odorranalectin, *PD* Parkinson’s disease, *PEG-PCL* PEG-poly (ɛ-caprolactone), *PEG-PLA* PEG-polylactic acid, *PEG-PLGA* PEG-poly(lactic-glycolic acid), *PLA* polylactic acid, *PVP* poly(*N*-vinyl pyrrolidone), *SP* substance P, *TRP* thyrotropin-releasing hormone, *VIP* vasoactive intestinal peptide, *WGA* wheat germ agglutinin

Most of the in vivo studies involving the use of peptides loaded onto nanocarriers were aimed at the treatment of Alzheimer’s disease (AD). The goal of the early studies, performed in 2007, was to investigate the potential of WGA-functionalized PEG-PLA NPs to enhance the transport of the vasoactive intestinal peptide. The results showed that the peptide concentration in the brain was 7 times higher when associated to the NPs as compared to the free peptide [141]. A peptide named humanin derivative was associated to PEGylated cubosomes (93 nm) functionalized with OL peptide. The results obtained by fluorescence imagining in an AD rat model indicated that the OL functionalization led to a 2.6-fold increase in the access of cubosomes to the brain. This increased brain delivery translated into a significant enhancement of the therapeutic effect [151]. The NAP neuropeptide was loaded into Lf-functionalized PEG-PCL NPs, of 90 nm, and the N-to-B transport was assessed by fluorescence imagining in an AD rat model. The results indicated that the Lf functionalization of the NPs led to their enhanced transport to the brain, which correlated with a deceleration of memory loss [155]. Recently, some authors reported that simple nanogels made of PVP with a particle size of 90 nm and negative surface charge could be used for the enhanced delivery of insulin to the brain. The accumulation of fluorescent-labelled insulin in the brain was visualized by fluorescence microscopy and its activity was measured by Akt activation levels, in an AD mice model [119]. Although the authors hypothesized different potential mechanisms for the transport of the nanogels across the N-to-B barriers, the study did not provide direct and quantitative evidence of the mechanisms involved in the transport.

Some peptides have also been explored for the treatment of Parkinson’s disease (PD). For example, the urocortin peptide, capable of restoring nigrostriatal function, was loaded into PEG-PLGA NPs functionalized with OL and administered intranasally in a PD model. The results reported a six- and threefold increase in the therapeutic effect in comparison with intranasal administration of the free peptide or the unmodified NP, respectively [150]. A peptide named substance P, intended to protect dopamine neurons from neurotoxicity, was loaded into gelatin-cored nanostructured lipid carriers (NLCs) (170 nm, negative surface charge) and assayed in a PD rat model. The results obtained following nasal administration, showed an increase in the efficacy, assessed by quantifying rotational behaviour and levels of proteins related to PD [114].

Some peptides associated to nanocarriers have also been proposed for the treatment of epilepsy. For example, the anticonvulsant thyrotropin-releasing hormone (TRH) was associated to WGA-functionalized PLA NPs (100 nm) and labelled with a fluorescent marker. Following nasal administration to epileptic rats, the NPs were found to be able to reach the brain and suppress seizures [110]. The NR2BPc peptide, of potential interest for the treatment of ischemia and prevention of strokes, was loaded into WGA-functionalized PLA-PEG NPs (140 nm, and negative surface charge). Following intranasal administration of this formulation to an ischemic rat model, a significant reduction in the size of the infarcted area was reported [144].

The peptide leucine-enkaphalin, associated to trimethyl chitosan NPs (> 400 nm, positive charge) was explored for pain relief purposes. The peptide was labelled with a fluorescent tag for the evaluation of its biodistribution. Following intranasal administration, an increased antinociceptive effect was reported, along with higher brain accumulation for the nanoformulation, as compared to the free drug [107].

Finally, cyclosporine-A (CsA), which has been reported to exhibit neuroprotective properties, has been formulated as a nanoemulsion (270 nm and a positive surface charge) made of flax-seed oil, also known for its neuronal regulating characteristics. A significant brain accumulation of CsA was observed following intranasal administration, a fact that was attributed to a direct N-to-B uptake [108].

### Proteins

The nanotechnological strategies adapted for proteins are similar to those used for peptides (Table 4) [171, 172]. The most frequently tested protein as a cargo of different nanocarriers is basic fibroblast growth factor (bFGF), which depicts neuroprotective effects in different brain-related diseases. For example, bFGF associated to functionalized STL-PEG-PLGA NPs (120 nm and negative surface charge) was administered intranasally in an AD mice model. The results showed that loaded NPs enhanced 1.5 times the AUC of radio-labelled-bFGF compared to free protein, and the modification with the targeting ligand further increases the value of AUC up to 3 times more, as described above [147]. The same protein, bFGF encapsulated into gelatin NLC (128 and negative surface), containing phospholipids, cholesterol, and Poloxamer 118, was tested in an ischemia rat model. The results highlighted a 1.5 times greater protein accumulation in different brain areas, when compared with intravenous administration, as well as enhanced therapeutic response [173]. The same nanocarrier was used to deliver bFGF for the treatment of PD. The results showed high protein levels in different areas of the brain, including the olfactory bulb and striatum, and enhancement of their therapeutic effect after intranasal administration in a PD rat model, as compared with free protein and intravenous administration of the nanoencapsulated protein [115].

**Table 4:** Overview of selected nanocarrier systems for proteins N-to-B delivery

Nanosystem	Peptide cargo	Disease	Size (nm)	Z-Pot (mV)	Targeting molecule	Animal model	Ref
PEG-PLGA NPs	bFGF	AD	120	− 32	STL	Rat	[147]
Gelatin NLCs	bFGF	Ischemia	128	− 15	-	Rat	[173]
Gelatin NLCs	bFGF	PD	172	− 28	-	Rat	[115]
CS NLCs	GDNF	PD	137	+ 30	-	Rat	[137]
CS NLCs	hIFG-1	-	114	+ 28	-	Mice	[136]
PEG-PLA polymersomes	BDNF	Neuro-inflammation	270	− 20	-	Mice	[109]

*AD* Alzheimer’s disease, *BDNF* brain-derived neurotrophic factor, *bFGF* basic fibroblast growth factor, *CS* chitosan, *GDNF* glial cell-derived neurotrophic factor, *hIFG-1* human insulin-like neurotrophic growth factor-I, *NLC* nanostructured lipid carriers, *NPs* nanoparticles, *PD* Parkinson’s disease, *PEG-PLA* PEG-polylactic acid, *PEG-PLGA* PEG-poly(lactic-glycolic acid), *STL* *Solanum tuberosum* lectin

The glial cell-derived neurotrophic factor (GDNF), also applied to Parkinson’s disease, has been entrapped in chitosan NLCs, using a combination of solid and liquid lipids. Their intranasal administration in PD model rats resulted in a meaningful behavioural improvement [137]. These authors fluorescently labelled these nanosystems for studying the N-to-B transport of human insulin-like neurotrophic growth factor-I (hIFG-I). The fluorescent images indicated a high accumulation in the brain, with diffusion and penetration into different brain areas. Moreover, NLCs were strongly detected in the olfactory tract, a result that was attributed to the mucoadhesive properties of chitosan [136].

The brain-derived neurotrophic factor (BDNF), encapsulated into PEG-PLA polymersomes (270 nm and a negative surface charge) was co-administered with simvastatin intranasally in a neuro-inflammation mice model. The results showed a significant reduction of cytokine levels and microglial activation in different brain areas when both drugs are co-administered, compared to single-drug nanoencapsulated administration [109].

### Monoclonal antibodies

Among the different protein-based therapies, monoclonal antibodies (mAbs) have gained particular attention over the past decades, resulting in an increasing amount of therapeutic antibodies on clinical trials and even in the market [174–176]. Interestingly, FDA recently approved the use of a monoclonal antibody, aducanumab (marked as Aduhelm), for the treatment of AD, thus paving the way to further development of antibody-based treatments for CNS conditions [7, 8]. Despite the interest of mAbs for the treatment of neurological diseases, to our knowledge, the use of nanotechnology for the N-to-B delivery of these molecules has not been explored. The only report we have found makes use of the mAb anti-EPH3 as a targeting ligand attached to PLGA NPs coated with trimethyl chitosan. This approach relies on the fact that anti-EPH3 targets a membrane receptor overexpressed in the stroma and vasculature of gliomas. The NPs were loaded with temozolomide and administered intranasally in a glioma rat model. Higher brain accumulation of NPs was determined by fluorescence imaging after they were functionalized with anti-EPH3 antibody, compared with unfunctionalized NPs. Moreover, median survival time and apoptosis of glioma cells was significantly enhanced upon treatment with the functionalized NPs [177].

### RNA (siRNA, miRNA)

Among the different types of biomolecules, RNA constructs may be one of the more challenging to be delivered through N-to-B pathways [178, 179]. In addition to the challenges described for the delivery of peptides and proteins, the RNA molecules need to reach the intracellular space in order to execute their action. To achieve this, RNA molecules must remain associated to the NPs until reaching the intracellular target and the NPs must be able to cross intact the N-to-B barriers (as shown in Fig. 5) [180, 181]. Considering all these requirements, different nanocarriers had been designed for the efficient N-to-B transport of nucleic acids (Table 5).

**Table 5:** Overview of selected nanocarrier systems for RNA N-to-B delivery

Nanosystem	RNA cargo	Disease	Size (nm)	Z-Pot (mV)	Targeting molecule	Animal model	Ref
PEG-PCL nanomicelles	FAM-siRNA	-	50	+ 10	Tat	Rat	[124]
PEG-PCL nanomicelles	siRNA Raf-1	Gioblastoma	160	+ 9	Tat	Rat	[125]
CS NPs	siRNA Gal-1	Glioblastoma	141	+ 32	-	Mice	[133, 182]
PEG-PCL nanomiclles	siRNA TNF-α	Ischemia	62	+ 19	Tat	Rat	[126]
CS NPs	siRNA HTT	HD	104–205	+ 43–55	-	Mice	[113]
PEG-PLGA NPs	miR-124	Ischemia	204	-	RVG29	Rat	[157]
PEG-PLA NPs	miR-132	AD/Ischemia	191	-25	WGA	Mice/ Rat	[143]
PEG-PGA—r8-C12 NCXs	miR-132	AD	96	+ 4	-	Mice	[130]
Au-Fe_2_O_3_ NPs	miR-100 and antimiR-32	Glioblastoma	50	+ 4	T7	Mice	[134]

*AD* Alzheimer’s disease, *Au-Fe*_*2*_*O*_*3*_ gold-iron oxide, *CS* chitosan, *FAM* 6-carboxyfluorescein-aminohexyl, *Gal-1* Galectin-1, *HD* Huntington’s disease, *HTT* huntingtin, *NCXs* nanocomplexes, *NPs* nanoparticles, *PEG-PCL* PEG-poly (ɛ-caprolactone), *PEG-PGA* PEG-polyglutamic acid, *PEG-PLA* PEG-polylactic acid, *PEG-PLGA* PEG-poly(lactic-glycolic acid), *r8-C12* octaarginine-lauric acid, *TNF-α* tumour necrosis factor-α

Among the different types of RNAs, siRNA has received the greatest attention for the treatment of neurological pathologies and, hence, for the N-to-B delivery. One of the first nanocarriers developed for the delivery of siRNA molecules (including FAM-siRNA, siRNA Raf-1 and siRNA TFN-α) was the Tat-modified PEG-PCL nanomicelles (size of 50–160 nm and positive surface charge) [124–126]. This nanocarrier could efficiently transfer the FAM-siRNA molecules into the olfactory and trigeminal nerves [124]. Further studies with the same nanosystems involved the encapsulation of both siRNA Raf-1 and camptothecin (CPT) for the treatment of glioblastoma. The in vivo results showed a significant accumulation of fluorescent siRNA in the brain, which translated into a greater survival time and tumour reduction [125]. Similarly, siRNA Gal-1-loaded chitosan NPs (140 nm and positive surface charge) were administered intranasally in a glioblastoma mice model. The results showed an overall increase in the survival rate of mice, affecting the tumour microenvironment composition, polarization of macrophages, or tumour vasculature. Moreover, the combination of siRNA Gal-1-loaded chitosan NPs with temozolomide or PD-1 blocking resulted in a significant synergic effect, increasing survival rate [133, 182].

The use of siRNA-loaded NPs for the treatment of a neurodegenerative disease, i.e. Huntington’s disease (HD), has also been explored. siRNA anti-huntingtin (HTT)-loaded chitosan-modified NPs were administered intranasally in a HD mice model. Hydrophobic modifications of siRNA led to a minor silencing effect in some brain areas, whereas hydrophobic modified siRNA-loaded chitosan NPs led to a reduction in the HTT mRNA and protein levels [113].

Finally, siRNA TNF-α-loaded Tat- modified PEG-PCL nanomicelles (62 nm and positive surface charge) were administered intranasally in an ischemic stroke mice model, and the result of this treatment was a reduction of the infarcted area [126].

Different nanocarriers had been explored for the delivery of different types of miRNA for the treatment of diverse CNS conditions. For example, PEG-PLGA NPs, chemically modified with RVG29, were developed for the intranasal delivery of miR-124 in an ischemia rat model. Interestingly, both targeting ligand and PEG chain were identified as key elements for the successful delivery of miRNA to the brain, significantly improving the modulation of its target mRNAs and the neurobehavioural score along with a reduction in the infarcted brain area [157]. In a different study, the potency of miR-132 for the treatment of AD and ischemia was evaluated in mice and rats, respectively. miR-132 was complexed with spermidine, and the resulting complex was encapsulated onto PEG-PLA NPs (191 nm and negative surface charge) chemically modified with WGA. In an AD mice model, significant improvement in terms of behaviour as well as decreasing Aβ levels were reported. Moreover, enhanced neurobehavioural score and reduction on infarcted brain area were achieved on an ischemic rat model, proving the promising effect of miR-132 for the treatment of different CNS conditions [143].

An enveloped nanocomplexes (ENCPs) technology was developed in our lab, consisting on miRNA-132 complexed to r8-C12 and enveloped with PEG-polyglutamic acid (PGA) (96 nm, neutral surface charge). This formulation resulted in a significant modulation of mRNA targets in the olfactory bulb and the hippocampus after intranasal administration [130].

Combination of miR-100 and antimir-32 had shown potential for the treatment of glioblastoma. In this case, both miRNAs have been incorporated onto gold-iron oxide NPs, functionalized with T7 peptide and β-cyclodextrin chitosan. The combination of intranasal administration of loaded gold-iron oxide NPs with systemic administration of temozolomide (TMZ) led to an increase in the survival time and magnetic resonance imaging properties, showing potential as a theragnostic agent [134].

In conclusion, different nanocarriers have been used that showed efficient nucleic acid delivery to the brain after intranasal administration. Overall, the tendency observed is that functionalized nanocarriers with targeting ligands or CPPs exhibit a greater performance compared to the non-functionalized ones.

## Conclusion and future perspectives

Neurological disorders and the possible therapeutic strategies that would address them have been a subject of increased interest over the last decades. Among the different possibilities to successfully access the CNS, the main difficulty in this line of study, the use of nanocarriers has shown some potential for the direct N-to-B transport. Since the first successful attempt reported by Gao et al. in 2007, a variety of nanotechnological approaches have been developed and tested in animal models [141]. These approaches could be particularly beneficial for the N-to-B delivery of biomolecules, given that their access to the brain following systemic administration has proved extremely difficult so far.

In order to develop potential nanocarriers for the N-to-B transport of biomolecules, it is fundamental to take into consideration the different barriers that must be overcome and the way that nanocarriers can be specifically designed to overcome them. With regard to the physicochemical characteristics of the nanosystems, there is certain evidence that a small particle size correlates with an improved delivery of cargo from the nasal cavity to the brain. The majority of the nanocarriers explored present a mean diameter between 50–200 nm; however, the smaller ones have, apparently, a greater chance to be transported to the brain. On the other hand, the influence of the surface charge in the N-to-B performance of nanocarriers is not that clear, although the majority of the nanocarriers reported to date exhibit a negative or neutral charge. Finally, the surface composition of the nanocarriers has an impact on their N-to-B transport. Of note, the use of permeation-enhancing molecules and the addition of PEGylated compounds have been reported to impact this transport. Moreover, the combination of these strategies with targeting ligands has also been reported as a promising strategy.

However, despite these promising findings and studies, none of these technologies intended for N-to-B delivery of biomolecules has reached the clinical development phase. This is understandable if we take into account the reduced number of in vivo studies performed to assess the value of these technologies. Moreover, an important drawback found in most of the relevant articles reporting in vivo results is the use of fluorescence imaging to confirm the presence of the drug in the brain after nasal administration. This type of imaging can be, in some cases, misleading and not a reliable quantitative approach for the determination of the amount of cargo delivered to the brain. Only a few studies have done a real quantification of the drug in different areas of the brain, to demonstrate the presence of the drug, and further studies need to be performed in order to confirm an adequate delivery. Importantly, it is not only fundamental to ensure an adequate transport of the macromolecules to the desired brain areas, but to ensure the maintenance of their functionality. In this regard, it would be critical to generate additional knowledge on the assessment of the pharmacokinetics and biodistribution of the therapeutic molecules in addition to their biological effect.

In conclusion, N-to-B transport of biomolecules has been shown to be a potential alternative for the delivery of drug-loaded nanocarriers in sufficient amount to depict a therapeutic effect for the treatment of different neurological conditions. However, the application in the clinic of a combination of nanotechnology and N-to-B delivery is still at very early stages. To facilitate this promising translation, more studies to elucidate the suitable characteristics that a nanosystem must possess to be a successful carrier must be performed in more conclusive animal models, such as macaques. Furthermore, more conclusive data is needed regarding the pharmacodynamics and pharmacokinetics of the different pathways followed, as well as the biodistribution of the biomolecules in the brain.

## Data Availability

Not applicable.
